# AI-orchestrated platelet dynamics analyzers: a paradigm shift in thrombotic risk mitigation for internal medicine

**DOI:** 10.1097/MS9.0000000000003816

**Published:** 2025-08-28

**Authors:** Muhammad Talha, Maliha Khalid, Aminath Waafira

**Affiliations:** a King Edward Medical University, Lahore, Pakistan; b Jinnah Sindh Medical University, Karachi, Pakistan; c Department of Medicine, The Maldives National University, Malé, Maldives

*To the Editor,*

The evolution of artificial intelligence (AI) in medicine has provided radical new models for diagnosis and therapy for thrombotic disorders. Internal medicine is challenged in individualizing risk assessment and timely intervention, while recent advances in AI-enabled platelet dynamics analysis offer a most appealing solution to thrombotic risk reduction – a concept that has matured rather significantly in 2025.

Thrombosis has been one of the major causes contributing to morbidity and mortality across the globe. This includes venous thromboembolism (VTE), which, by itself, leads to tremendous numbers of hospitalizations and deaths each year^[1]^. Conventional approaches for risk stratification possess definite value but often suffer from a certain level of inaccuracy as they do not offer proper applicability and resolution for patient-specific prediction. This limitation becomes particularly acute in very complex situations like acute coronary syndrome (ACS) and surgical environments, where dynamic platelet behavior holds key importance in determining thrombotic outcomes^[1,2]^. The cell-based platelet dynamics analyzers basically combine imaging, machine learning, and real-time analytics closely tailored to assessing the behavior of the platelets and getting insights into their functions, unlike conventional measures, such as light transmission aggregometry, which offer only generalized, time-averaged information on platelet aggregation and interactions^[3]^. The confluence of AI, advanced imaging, and real-time analytics would now allow for unprecedented resolution and speed in tracking and interpreting platelet dynamics in real time.

This innovation has recently been demonstrated through work done at the University of Tokyo. With the help of frequency division multiplexed (FDM) microscopy and advanced artificial intelligence, this technique presents the first real-time tracking of platelet aggregation in which thousands of images are recorded per second. Machine learning was then applied in distinguishing single platelets from aggregates and even white blood cell interactions. When applied to over 200 patients, it was found that those suffering from acute coronary syndrome had significantly greater amounts of platelet aggregates compared to those with chronic symptoms. This emphasizes how AI-dependent analyzers would be a real-time risk assessment tool^[4]^. Such systems would not only improve the quality of diagnostic assessment and help with patient-tailored antiplatelet therapy by providing clinicians with patient-specific actionable data. AI-based tools for real-time risk assessment have recently become a focus of study. They do more than assist in the accurate diagnosis; they also facilitate the personalization of antiplatelet therapy through individualized, actionable data^[5]^.

Further, artificial intelligence algorithms are increasingly being used for risk stratification, early detection, and management of thrombotic events beyond imaging. Machine learning models outperform the traditional models in identifying high-risk candidates for VTE prophylaxis, especially in the postsurgical population. These models amalgamate various sources of data, such as laboratory results, imaging, and clinical notes, to provide good predictions and timely interventions^[1]^. In addition, the ability of AI to analyze genetic data and molecular data will enable us to understand the disorders of coagulation factors better and even diagnose these significantly earlier with much more targeted options for treatment^[6,7]^.

Despite these developments, there are still difficulties in the way. There are still barriers to the acceptable clinical use of AI tools for thrombotic risk mitigation that include data quality, model interpretability, and the necessity for multidisciplinary collaboration^[1,8]^. To ensure fair distribution of health AI systems, patient privacy risks posed by potential data breaches or algorithmic biases resulting in uneven model performance across distinct populations must be ethically addressed^[7]^. Inaccurate risk predictions due to biased training data may render the affected groups underrepresented during validation and fairness audits. Research and stakeholder engagement must be sharpened to bridge the gap between AI innovation and routine clinical practice^[8,9]^.

To conclude, AI-controlled analyzers for platelet dynamics are paradigms for internal medicine of the future, 2025. Using fast imaging, machine learning, and predictive analytics, real-time monitoring, very accurate risk stratification, and personalized mitigation of thrombotic risk are provided. Instead, the medical community should strive to embed AI tools into clinical pathways, set aside funding for robust validation studies, and encourage interdisciplinary partnerships, so the full great benefits can be harvested. At best, these technologies will promise an improvement in patient outcomes and change the standards of care for thrombotic disorders. Our work is in line with the TITAN Guidelines on the need for transparency in AI use in healthcare^[10]^.

## Data Availability

None.
